# New Improved Fractional Order Differentiator Models Based on Optimized Digital Differentiators

**DOI:** 10.1155/2014/741395

**Published:** 2014-02-06

**Authors:** Maneesha Gupta, Richa Yadav

**Affiliations:** Netaji Subhash Institute of Technology, Sector 3, Dwarka, New Delhi 110078, India

## Abstract

Different evolutionary algorithms (EAs), namely, particle swarm optimization (PSO), genetic algorithm (GA), and PSO-GA hybrid optimization, have been used to optimize digital differential operators so that these can be better fitted to exemplify their new improved fractional order differentiator counterparts. First, the paper aims to provide efficient 2nd and 3rd order operators in connection with process of minimization of error fitness function by registering mean, median, and standard deviation values in different random iterations to ascertain the best results among them, using all the abovementioned EAs. Later, these optimized operators are discretized for half differentiator models for utilizing their restored qualities inhibited from their optimization. Simulation results present the comparisons of the proposed half differentiators with the existing and amongst different models based on 2nd and 3rd order optimized operators. Proposed half differentiators have been observed to approximate the ideal half differentiator and also outperform the existing ones reasonably well in complete range of Nyquist frequency.

## 1. Introduction

Fractional calculus (FC) is an unusual topic that still propels much novel advancement with the growing interest of many researchers in different domains of science and engineering as it provides an additional authentication factor to all the fractional order systems by generalizing integrals and derivatives of any arbitrary order. Apart from engineering sciences, FC has also been used in other distinct research areas including statistical modelling, mechanical system analysis, control, automated control, thermal systems, electromagnetism, image processing, radio engineering, and signal processing. The frequency response of ideal fractional order differ-integrator (*s* ^±*α*^) is

(1) $$\begin{array}{c} H_{\text{fractional}}\left(j\omega \right)={\left(j\omega \right)}^{\pm \alpha }, \end{array}$$

where $j=\sqrt{-1}$ and *ω* gives angular frequency in radians. Variable *α* defines the order of fractional order differ-integrators and its value lies between 0 and 1.

In literature, volumes of work have been found in different designing methods of fractional order integrators (FOIs) and fractional order differentiators (FODs) which strengthen the grasp of this exotic field over not only traditional filter design methods but also in a gamut of newly advanced science and engineering network development methods. Unambiguously, Newton-Cotes integration rules have been ruling the trajectory of almost all the crucial design procedures of digital integrators since last two decades [1–5]. Another significant turn that came in the design procedure of operators was with the evolution of linear interpolation of the existing integrators going hand in hand with linear programming (LP) optimization, which resulted in the design of different efficient recursive digital integrators [6–10].

As far as fractional domain is concerned, there are many distinct approaches which have been regularly used in practice for designing FOIs and FODs. One approach which is vastly used by many researchers, involve interpolation of two existing digital integrators by linear interpolation and later discretization of the resulting model by either direct discretization [11–16] or by indirect discretization [17–20]. Second step in the procedure of these discretization streams can be experimented with variations in the order of fractional operator (value of *α* = 1/2,1/3,1/4) as well as order of truncation (value of fractional order (FO)) resulting in one-half, one-third, and one-fourth integrators and differentiators for different orders [21–25].Fractional calculus has also tasked itself with different iterative and noniterative approximation techniques *vis-á-vis* Laguerre approximation [26], Pade, Prony and Shank's approximations [27], Ostaloup's approximation [28], Newton Raphson method [29], and iterative signal modeling technique of Steiglitz-McBride method [30] for spinning off its domain to different sectors.

The fact which has remained in the backdrop is the simple case of optimization of an operator (*s*-to-*z* transform) before converting it into fractional order by some discretization scheme. This paper attempts to push an operator which is to be discretized near boundaries of an optimal solution before its discretization, because every minute dispensation in properties of this *integer* order operator will directly propel into its *noninteger* order counterpart. A unique combination of an optimized operator and an accurate approximation technique definitely configures into an efficient fractional operator. Recent acceleration in the trend of optimization using different evolutionary optimization algorithms (EAs), namely, simulated annealing (SA) [31], pattern search, genetic algorithm (GA) [32], particle swarm optimization (PSO) [33, 34], and hybrid evolutionary algorithms [35–40], can give an easy answer to this simple yet unaddressed scenario.

Authors in this brief have unveiled efficient application of three vastly popular optimization techniques, namely, PSO, GA, and a hybrid optimization algorithm, namely, PSO-GA hybrid evolutionary algorithm, in which PSO and GA have been crossed in parallel. Optimization of 2nd and 3rd order operators backed by expertise of these three techniques has been presented here. The results of magnitude responses, phase responses, and relative magnitude errors in decibel (RME (dB)) for the proposed fractional differentiators have been plotted in MATLAB. 3rd order digital differentiators optimized by PSO-GA hybrid optimization algorithm clearly excel existing Al-Alaoui optimized 3-segment operator [1] with RME (dB) of ≤−35 dB in 1.77 ≤ *ω* ≤ 2.80 *π* radians and Third order PSO optimized differentiators excel all the other proposed operators with RME (dB) of ≤−50 dB in almost complete range. Second order PSO and PSO-GA hybrid optimized differentiator outperform recently published 2nd order PSO optimized differentiator [32] with RME (dB) of −70 dB and −60 dB in frequency ranges of 0 ≤ *ω* ≤ 0.615 *π* radians and 1.4 ≤ *ω* ≤ 1.8 *π* radians, respectively, whereas PSO-GA hybrid optimized performs better in range of 2.22 ≤ *ω* ≤ 2.87 *π* radians of full spectrum.

Later these optimized operators are discretized by CFE of indirect discretization scheme, for finding half differentiators of different orders. Proposed FODs based on 2nd order optimized operators have been proved to be more effective than recent half differentiator given by Leulmi and Ferdi [30] especially in higher frequency range with RME (dB) of the order of ≤−52 dB over 2.15 ≤ *ω* ≤ 3 *π* radians and linear phase responses in almost complete frequency range. FODs based on all 3rd order optimized digital operators have been observed to have linear phases and RME (dB) of ≤−27.92 dB over 0.77 ≤ *ω* ≤ 1.7 *π* radians and ≤−36.26 dB over range of 2.1 ≤ *ω* ≤ 3 *π* radians when FO is 3 and ≤−26.47 dB over the range of 0.42 ≤ *ω* ≤ 1.9 *π* radians of complete range when FO is 6. In both cases FODs based on digital differentiators optimized by PSO-GA hybrid EAs perform superior to those based on PSO and GA optimized operators.

The paper is organized as follows: Section 2 deals with the brief description of PSO, GA, and PSO-GA hybrid algorithms. Procedures for the application of these three algorithms for optimizing differential operators have been suggested and the resultant 2nd and 3rd order optimized operators are presented in this section. These optimized operators have been discretized by indirect discretization using CFE technique of indirect discretization for deriving models of half differentiators in Section 3.  Section 4 presents the simulation results and performance discussion of comparisons of proposed FODs with their ideal responses and the existing ones.  Section 5 concludes the paper.

## 2. Evolutionary Algorithms for Optimizing 2nd and 3rd Order Digital Differential Operators

### 2.1. Outline of Basic Functionality of Different Algorithms

The emphasis on custom design and shrinking product cycles has led to an increased interest in optimization techniques [31–40]. In this paper, results of different evolutionary algorithms (EAs), namely, GA, PSO, and PSO-GA Hybrid algorithms, for finding optimal digital differentiators are derived and compared to show the superiority amongst each other.

**Figure 1:**
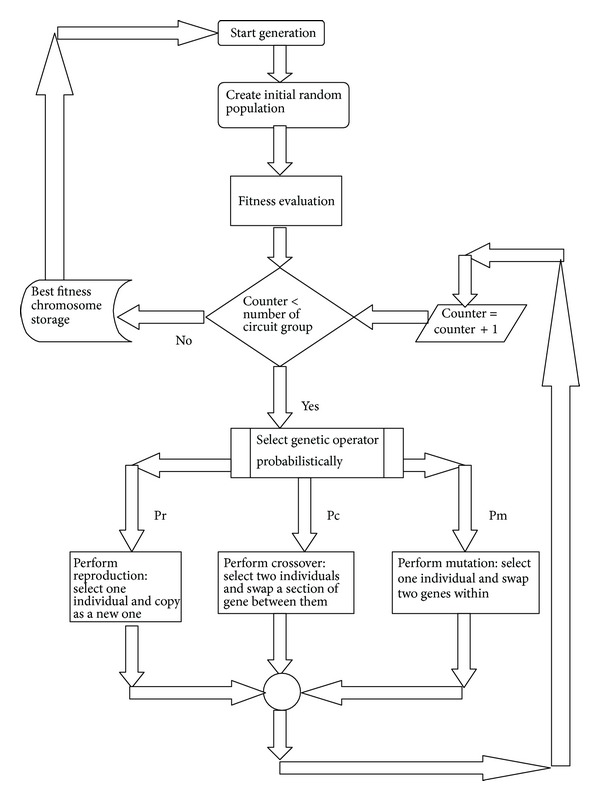
Flowchart of GA.

*Genetic Algorithm (GA)*. GA works on the heuristic search that mimics the process of natural evolution. In GA, a population of strings (called chromosomes) encodes candidate solutions (called individuals) to an optimized problem for finding a better solution. First the fitness function is defined over the genetic representation and then GA proceeds to initialize a population of solutions randomly which are later improved by repetitive application of different steps like mutation, cross over, inversion, and selection (see Figure 1).

*Particle Swarm Optimization (PSO)*. PSO is initialized with a population of random solutions and searches for optimal by updating generations. However, unlike GA, PSO has no evolution operators such as crossover and mutation. In PSO, the potential solutions, called particles, fly through the problem space by following the current optimum particles. Due to its advantages, PSO is not only suitable for scientific research, but also for engineering applications (seeFigure 2). In this paper for finding optimized operators, the parameters used for PSO are as follows: particle range = (−1, 1), learning factors (*c*1  and  *c*2) = 2, diversity factor = 1, maximum velocity factor = 0.5, swarm size = 200, and number of generations = 200.

**Figure 3:**
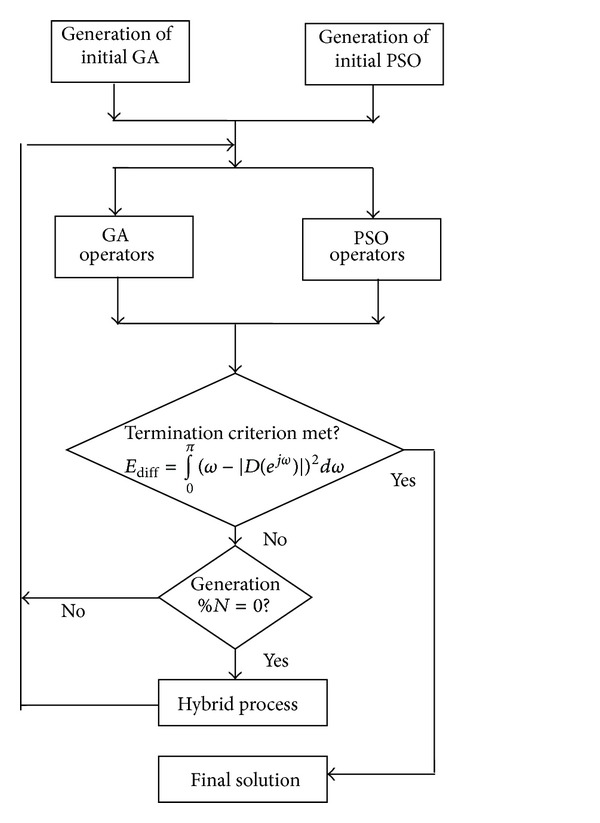
Flowchart of PSO-GA hybrid algorithm.

*PSO-GA Hybrid Algorithm*. This algorithm is obtained by crossing over PSO and GA algorithms by running these two systems simultaneously. In this P1, individuals from each system are selected for exchanging after designated iterations. Flowcharts of PSO-GA hybrid algorithm have been given below in Figure 3. Two parameters which mainly govern this algorithm are the number of particles selected for exchange between PSO and GA (P1) and the number of iterations after which exchange of individuals takes place between the two subsystems (N1).

### 2.2. Optimization of 2nd and 3rd Order Differentiators by Different EAs

In this paper when the code was run in C++ for 100 iterations, the best optimized (with least mean square error) results for 2nd and 3rd order proposed differentiators have been observed 200 generations using 200 particles. Intel Core 2 Duo CPU T6600 @2.20 GHz (installed memory (RAM) of 4 GB) has been used for simulations. The error function used here for finding optimal differentiators is

(2) $$\begin{array}{c} E_{\text{diff}}=\int_{0}^{\pi }{\left(\omega -\left|D\left(e^{j\omega }\right)\right|\right)}^{2}d\omega . \end{array}$$

Transfer functions (TF) of optimized digital operators were checked for stability and those which were found unstable were stabilized by pole reflection method. TF for proposed optimized 2nd and 3rd order differentiators have been given below in pole-zero form.

TF of 2nd order differential operator optimized by PSO algorithm is

(3) $$\begin{array}{c} G_{\text{PSO}\_2\text{nd}\_\text{opt}}\left(z\right)=\left(\frac{\left(1.156\left(z-0.9992\right)\left(z+0.5547\right)\right)}{\left(\left(z+0.1042\right)\left(z+0.6276\right)\right)}\right). \end{array}$$

TF of 3rd order differential operator optimized by PSO algorithm is

(4) GPSO_3rd_opt(z)  =((1.1553(z+0.38)(z+0.2522)(z−1))((z+0.1057)(z+0.2021)(z+0.503))).

TF of 2nd order differential operator optimized by GA is

(5) $$\begin{array}{c} G_{\text{GA}\_2\text{nd}\_\text{opt}}\left(z\right)=\left(\frac{1.2142\left(z-0.5788\right)\left(z+0.8210\right)}{\left(z+0.6403\right)\left(z+0.1913\right)}\right). \end{array}$$

TF of 3rd order differential operator optimized by GA is

(6) GGA_3rd_opt(z)  =(1.0802(z+0.0622)(z−0.9756)(z+0.1069)(z+0.5962)(z2−0.06085z+0.2721)).

TF of 2nd order differential operator optimized by PSO-GA Hybrid algorithm is

(7) $$\begin{array}{c} G_{\text{HYBR}\_2\text{nd}\_\text{opt}}\left(z\right)=\left(\frac{1.2192\left(z-0.8204\right)\left(z+0.7117\right)}{\left(z+0.7433\right)\left(z+0.1991\right)}\right). \end{array}$$

TF of 3rd order differential operator optimized by PSO-GA Hybrid algorithm is

(8) GHYBR_3rd_opt(z)  =(1.1583(z−0.9814)(z+0.4333)(z+0.3638)(z+0.6035)(z2+0.3945z+0.05831)).

**Figure 4:**
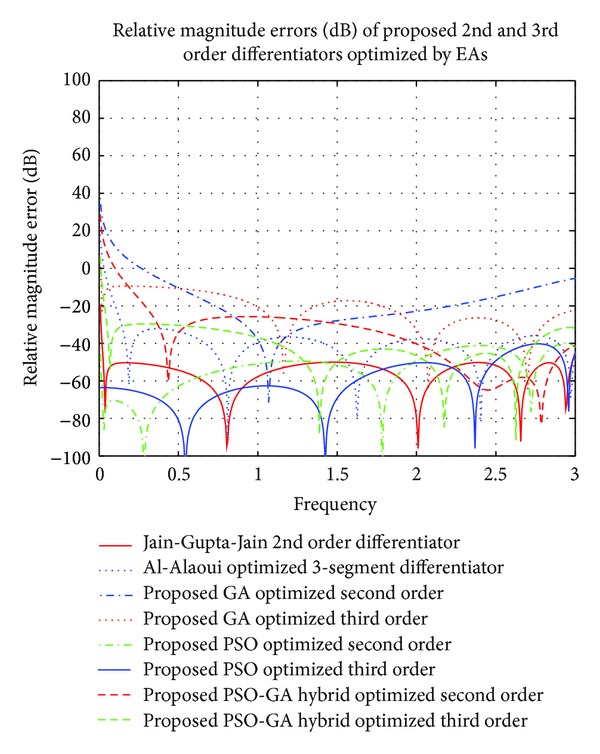
Comparison of relative magnitude errors (dB) of proposed optimized 2nd and 3rd order GA, PSO, and PSO-GA hybrid optimized digital differentiators with ideal and existing 2nd [32] and 3rd [1] order differentiators.

**Figure 5:**
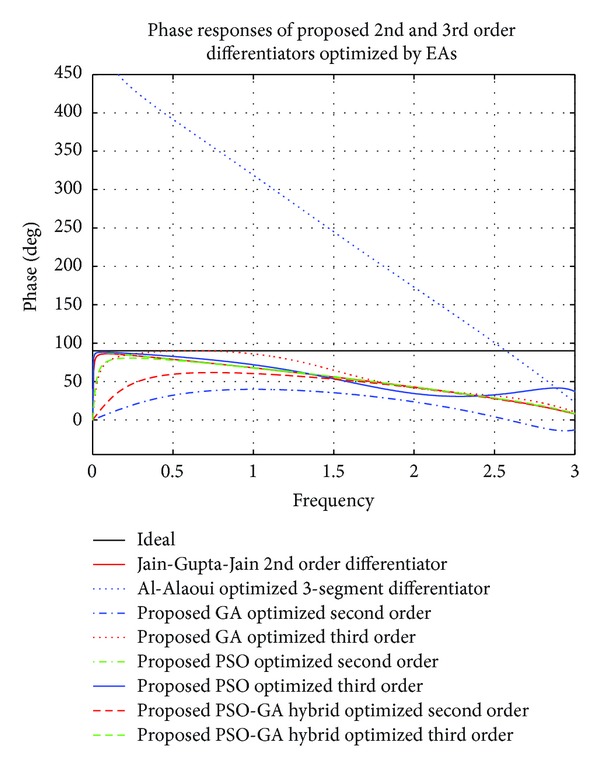
Comparison of phase responses of proposed optimized 2nd and 3rd order GA, PSO, and PSO-GA hybrid optimized digital differentiators with ideal and existing 2nd [32] and 3rd [1] order differentiators.

Comparison of responses of RME (dB) and phase responses of the proposed 2nd and 3rd order differentiators optimized here by different EAs, with recent 2nd order GA optimized digital differentiator given by Jain et al. in [32] and 3rd order Al-Alaoui optimized 3-segment differentiator [1], have been presented in Figures 4 and 5, respectively. All the differentiators are observed to satisfy stability criterion and show linearly decreasing phase responses. Proposed 2nd order PSO optimized operator, namely, *G*_PSO_2nd_opt_(*z*), outperforms Jain-Gupta-Jain differentiator [32] in frequency range over 0 ≤ *ω* ≤ 0.615 *π* radians and 1.4 ≤ *ω* ≤ 1.8 *π* radians with RME (dB) of −70 dB and −60 dB, respectively, whereas *G*_HYBR_2nd_opt_(*z*) gives better results than [32] in range of 2.22 ≤ *ω* ≤ 2.87 *π* radians. PSO optimized 3rd order differentiator, namely, *G*_PSO_3rd_opt_(*z*), clearly outperforms existing Al-Alaoui optimized 3-segment operator in almost complete range, but differentiator operator *G*_HYBR_3rd_opt_(*z*) given in (8) excels it over ranges of 1.04 ≤ *ω* ≤ 1.54 *π* radians and 1.77 ≤ *ω* ≤ 2.80 *π* radians of complete range with RME (dB) of ≤−35 dB.

### 2.3. Time Complexity of Different EAs in Optimizing Digital Differentiators

One factor that governs the success of any optimization algorithm and in most cases which is generally sidetracked by researchers is of its *time complexity*; that is, how different parameters affect the total time consumed in solutions for the best optimal results. Three main factors which directly affect the execution time are number of particles (*N*_*p*_), generation size (*N*_*g*_), and number of iterations for which code is allowed to run. Searching process in any random iterative process automatically terminates either after running for a fixed number of iterations which has been designated beforehand or when the aim of optimization of searching for the best optimal solution for the assigned fitness function is fulfilled. Complexity of the problem for which optimal solution is searched decides the time consumed in running the algorithm.

In this paper for the analyzing different EAs, number of iterations has been varied and the corresponding time taken in execution has been calculated for validating effectiveness of these algorithms. When the program in C++ is executed, the time taken for a single iteration of the program is less than 2 seconds whereas the total time of running the program for 100 iterations is 73 seconds in case of 3rd order differentiator and 56 seconds for 2nd order differentiator. The product of number of particles and number of generations (*N*_*p*_∗*N*_*g*_) has been varied from (50∗50) to (100∗100) and then to (200∗200) for different iterations and values of mean, median, and standard deviation have been registered for all the worst, average, and best results of 3rd order optimized differentiators (see Table 1). From this table, it is clear that PSO-GA hybrid technique performs better than PSO and GA algorithms by inheriting advantages of these otherwise efficient algorithms and also it was noticed that with the increase in values of *N*_*p*_, *N*_*g*_, and number of iterations the response of resultant differentiators come closer to the ideal response as mean, median and standard deviation values goes on decreasing with efficient minimization of error function. In this paper we have considered values of root mean square error.

As far as time complexity is concerned, the times consumed in finding optimal solutions for 3rd order differentiator operators by all the three EAs for different sets of (*N*_*p*_∗*N*_*g*_) have been also registered and are given below.

- GA: *T*_(50∗50)_: 4294964816 *μ*s, *T*_(100∗100)_: 4294965331 *μ*s, *T*_(200∗200)_: 4294964847 *μ*s.
- PSO: *T*_(50∗50)_: 4294962570 *μ*s, *T*_(100∗100)_: 4294962960 *μ*s, *T*_(200∗200)_: 4294965159 *μ*s.
- PSO-GA hybrid: *T*_(50∗50)_: 4294964457 *μ*s, *T*_(100∗100)_: 4294964254 *μ*s, *T*_(200∗200)_: 4294964956 *μ*s.

It is observed that for the population size 50 and setting number of generations to be equal to 50, the best results are given by PSO but with an increase in number of iterations and population size to 200 and PSO-GA hybrid algorithm gives comparable results.

## 3. Discretization of Proposed 2nd and 3rd Order Optimized Operators for Half Differentiator Models

The optimized differentiator operators derived in Section 2 are used as *s*-to-*z* transformations for CFE based indirect discretization scheme [17] for deriving half differentiators fitted in continuous-time domain. The resultant transfer functions of half differentiators (*s* ^±1/2^) based on 2nd and 3rd ordered optimized differentiators have been given in Tables 2 and 3, respectively. It can be clearly observed from pole zero form of TFs given in Tables 2 and 3 that all the proposed half differentiators are stable; that is, either these have all poles and zeros lying inside unit circle or interlaced along the line *z* ∈ (−1,1).

## 4. Simulation Results and Performance Discussion

### 4.1. Simulation Results of Proposed FODs Based on 2nd Order Differential Operators Optimized by PSO, GA, and PSO-GA Hybrid EAs

**Figure 6:**
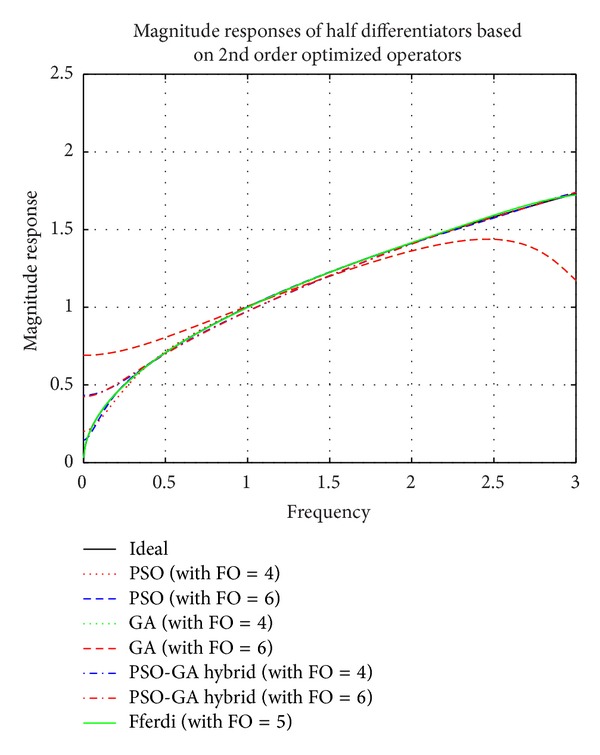
Comparison of magnitude responses of proposed half differentiators based on 2nd order optimized differential operators.

**Figure 7:**
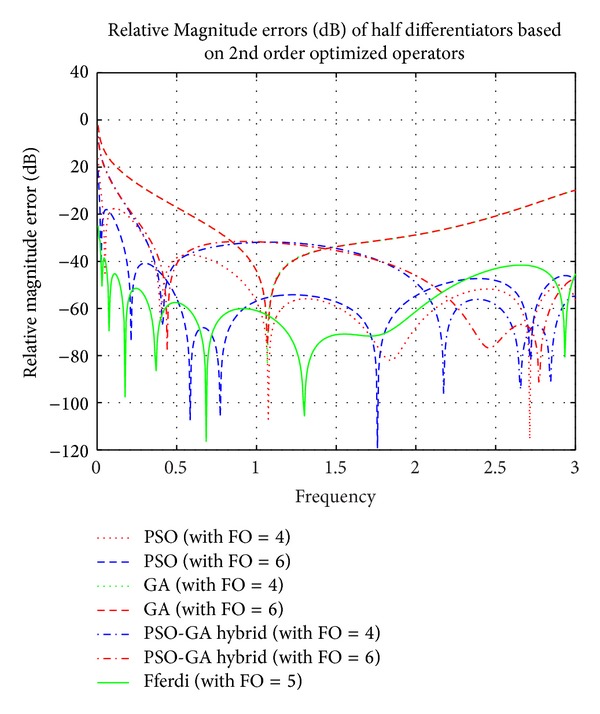
Comparison of relative magnitude errors (dB) of proposed half differentiators based on 2nd order optimized differential operators.

**Figure 8:**
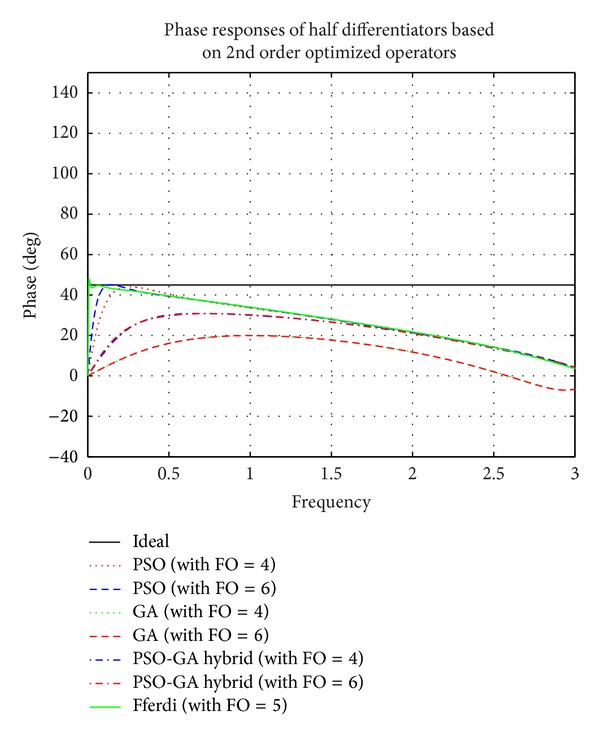
Comparison of phase responses of proposed half differentiators based on 2nd order optimized differential operators.

Simulation results of comparison of magnitude responses, RME (dB), and phase responses of half differentiators (see Table 2) based on different 2nd order optimized operators (given in (3), (5), and (7)) with the existing recent model based on a 2nd order new Simpson differentiator operator derived by Leulmi and Ferdi in [30] and ideal half differentiator have been presented in Figures 6, 7, and 8.

RME (dB) is given by

(9) $$\begin{array}{c} \text{RME}\left(\text{dB}\right)=20\;\log_{10}\left|\frac{k-k_{\text{approx}}}{k}\right|, \end{array}$$

where *k* is the magnitude of ideal value of the operator and *k*_approx_ is the value of the operator, that is, approximated.

It is observed that half differentiators based on 2nd order operator optimized by PSO algorithm show superior results as compared to other algorithms. Proposed FODs outperform Ferdi half differentiator in higher frequency region; that is, 2.09 ≤ *ω* ≤ 3 *π* radians with RME (dB) of −52 dB, and show linearly decreasing phase curve in almost complete frequency spectrum except in region near zero frequency. Forth half differentiator, namely, *G*_PSO_2nd_4_(*z*), closely follows Leulmi and Ferdi [30] differentiator and outperform it in ranges of 1.01 ≤ *ω* ≤ 1.25 *π* radians with RME (dB) well below ≤−60 dB and also in range of 1.7 ≤ *ω* ≤ 3 *π* radians of complete spectrum with error ≤−51.86 dB. *G*_PSO_2nd_6_(*z*) also excels [30] in different ranges *vis-á-vis *0.5 ≤ *ω* ≤ 0.93, 1.69 ≤ *ω* ≤ 1.83, and 2.3 ≤ *ω* ≤ 2.86 *π*. *G*_HYBR_2nd_4_(*z*) and *G*_HYBR_2nd_6_(*z*) show less than −32 dB RME (dB) value in full range of frequency.

### 4.2. Simulation Results of Proposed FODs Based on 3rd Order Differential Operators Optimized by PSO, GA, and PSO-GA Hybrid EAs

**Figure 9:**
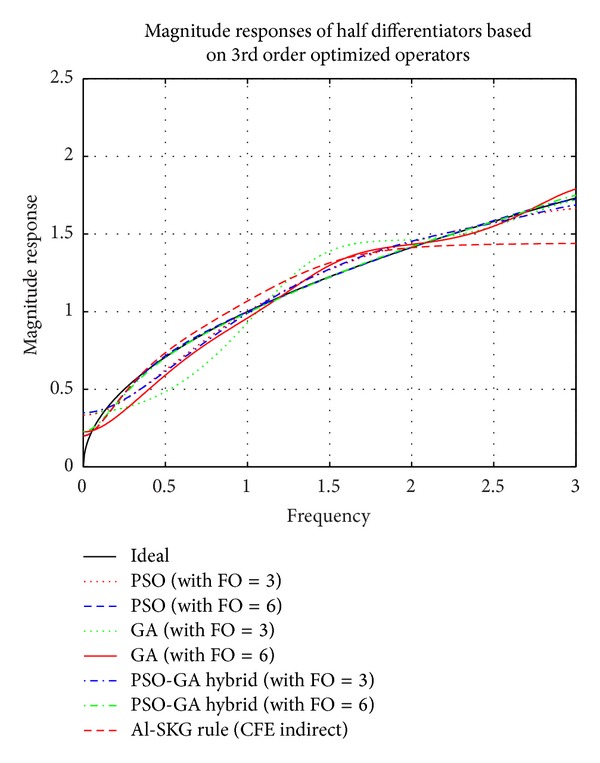
Comparison of magnitude responses of proposed half differentiators based on 3rd order optimized differential operators.

**Figure 10:**
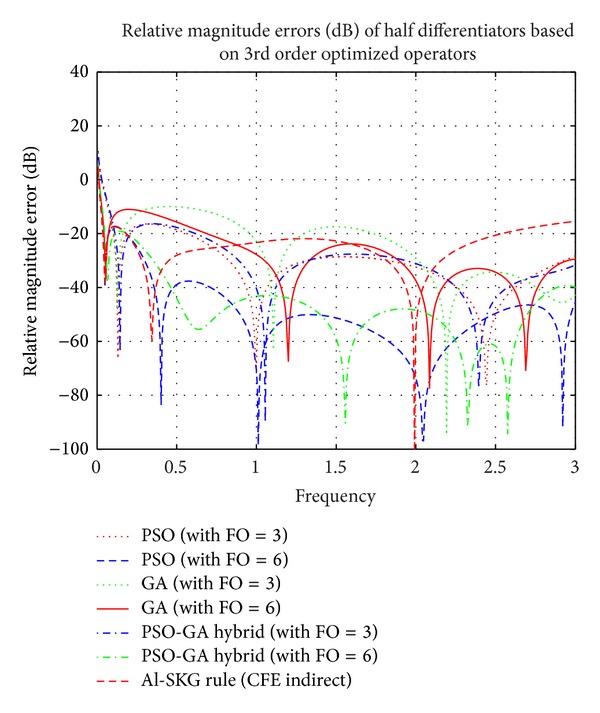
Comparison of relative magnitude errors (dB) of proposed half differentiators based on 3rd order optimized differential operators.

**Figure 11:**
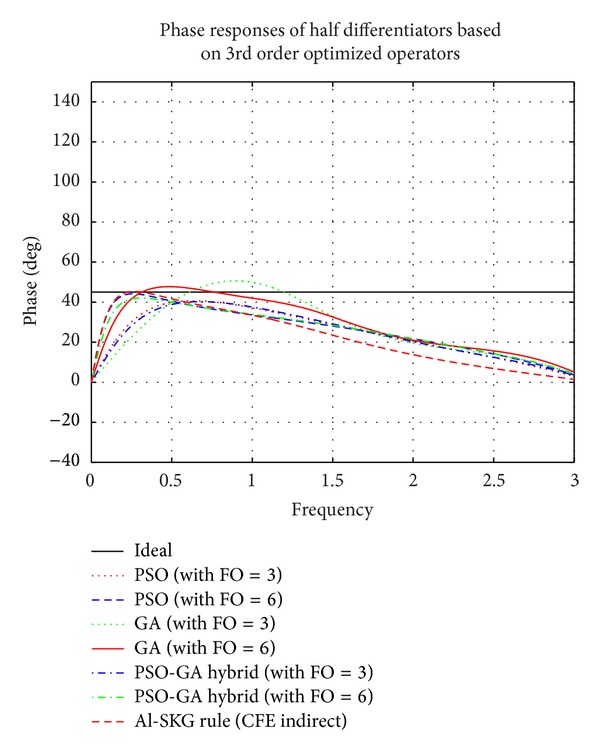
Comparison of phase responses of proposed half differentiators based on 3rd order optimized differential operators.

Figures 9, 10, and 11 show the plots of comparison of magnitude responses, RME (dB), and phase responses of proposed half differentiators (given in Table 3) based on proposed 3rd order optimized operators described by (4), (6), and (8), with Al-SKG operator (3rd order) based on half differentiators and ideal response. It can be clearly observed that 6th order half differentiator based on PSO-GA Hybrid optimized differentiator, namely, *G*_HYBR_3rd_6_(*z*), outperforms all other proposed half differentiators with RME (dB) of the order of ≤−40 dB in range of 0.46 ≤ *ω* ≤ 3 *π* radians of complete Nyquist frequency. Third order half differentiator *G*_HYBR_3rd_3_(*z*) outperforms Al-SKG rule based half differentiator with RME (dB) of ≤−27.92 dB in 0.77 ≤ *ω* ≤ 1.7 *π* radians and ≤−36.26 dB in 2.1 ≤ *ω* ≤ 3 *π* radians of complete range. All the FODs based on different 3rd order optimized operators show exactly linear phase curves in almost complete Nyquist frequency range. Simulation results of 6th order half differentiator based on PSO optimized operator and PSO-GA optimized operators, namely, *G*_PSO_3rd_6_(*z*) and *G*_HYBR_3rd_6_(*z*), respectively, lie in close proximity of each other but later one clearly outperforms the former in ranges of 0.42 ≤ *ω* ≤ 1.9 *π* radians. *G*_PSO_3rd_3_(*z*) and *G*_HYBR_3rd_3_(*z*) give comparable results and outperform half differentiators based on Al-SKG rule as well as FODs of GA optimized operator with RME (dB) of ≤−26.47 dB in range of 0.8 ≤ *ω* ≤ 3 *π* radians. *G*_GA_3rd_3_(*z*) and *G*_GA_3rd_6_(*z*) show comparatively superior results in higher frequency region.

## 5. Conclusion

This paper concludes that any fractional operator which can qualify as an efficient performance by attaining the heightened priority of less magnitude errors for different hardware applications embodies its base implicit in the quality of digital operator, which has been used as *s*-to-*z* transformation. So for obtaining efficient fractional operator one thing that should be of prime concern is that the properties of digital operators should not be left unmarked. This paper looks at this more precise subject by unpacking the discourse of possibilities to explain how properties of an efficiently optimized *s*-to-*z* transformation operator directly affect its fractional counterparts later derived by its discretization by exploring different optimization techniques for finding optimal 2nd and 3rd order differential operators. The simulation results of integer order operators have revealed the effectiveness of the proposed algorithms because of their low orders and less magnitude errors as compared to existing ones. Proposed 2nd, 3rd, 4th, 6^th^, and 9th order half differentiators based on these optimized digital differentiators show less RME (dB) of the order of −40 dB for almost the full band of Nyquist frequency. The proposed FODs show linear phase curves in almost full band of Nyquist frequency except near zero frequency regions.

The paper gives digital approximations to half-order differentiators obtained by discretization of optimal differentiators using different evolutionary algorithms (GA, PSO, and PSO-GA). The authors present a different approach for obtaining digital approximations of fractional-order operators. When this approach was compared with many other techniques used for the same purpose, it was observed that this one is more involved.

## Figures and Tables

**Figure 2:**
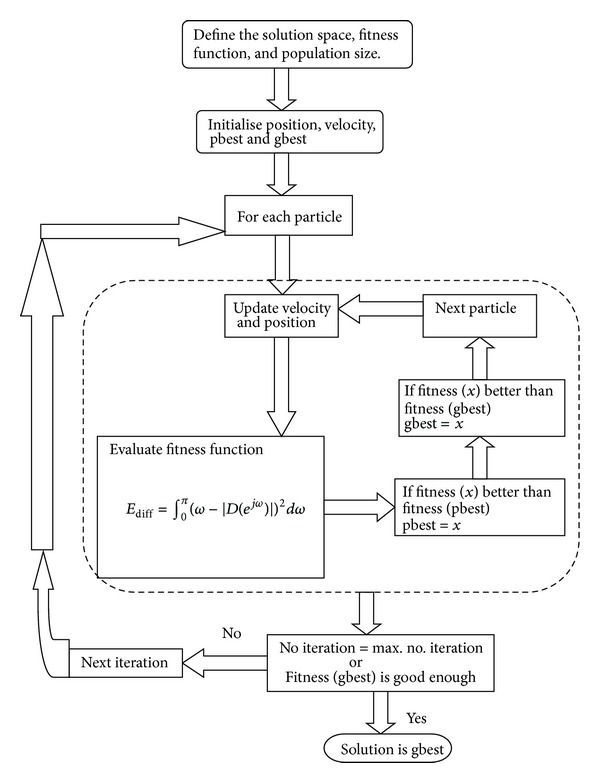
Flowchart of PSO algorithm.

**Table 1:** Comparative results for approximating 3rd order differentiator by different evolutionary optimization algorithms.

Evolutionary algorithm used	Number of populations used ∗ Number of generations involved								
	50∗50			100∗100			200∗200		
	Mean	Median	Standard deviation	Mean	Median	Standard deviation	Mean	Median	Standard deviation
GA	0.484438	0.280166	0.571391	0.174667	0.120752	0.176199	0.0887101	0.05331309	0.108299
PSO	0.210176	0.107856	0.369102	0.164112	0.0697475	0.281163	0.0906867	0.0293708	0.30069
PSO-GA hybrid	0.915405	0.1478565	0.737526	0.230096	0.069402	0.131617	0.0874599	0.02937085	0.0400587

**Table 2:** Mathematical models of half differentiators based on 2nd order optimized operators.

Mathematical models of half differentiators based on 2nd order optimized operator	
Algorithm	Transfer functions in pole-zero form
PSO	$G_{\text{PSO}\_2\text{nd}\_2}(z)=\left(\frac{1.0751\left(z-0.7416\right)\left(z+0.5604\right)}{\left(z+0.5869\right)\left(z-0.1823\right)}\right)$
	$G_{\text{PSO}\_2\text{nd}\_4}(z)=\left(\frac{1.0752\left(z-0.9026\right)\left(z-0.2927\right)\left(z+0.5788\right)\left(z+0.5566\right)}{\left(z-0.003298\right)\left(z+0.5633\right)\left(z+0.6072\right)\left(z-0.6391\right)}\right)$
	$G_{\text{PSO}\_2\text{nd}\_6}(z)=\left(\frac{1.0752\left(z-0.9494\right)\left(z-0.5919\right)\left(z+0.5936\right)\left(z+0.5649\right)\left(z+0.5556\right)\left(z-0.1105\right)}{\left(z-0.8067\right)\left(z-0.3424\right)\left(z+0.616\right)\left(z+0.5758\right)\left(z+0.5587\right)\left(z+0.04903\right)}\right)$

GA	$G_{\text{GA}\_2\text{nd}\_2}(z)=\left(\frac{1.1017\left(z-0.4323\right)\left(z+0.8014\right)}{\left(z+0.7299\right)\left(z-0.06811\right)}\right)$
	$G_{\text{GA}\_2\text{nd}\_4}(z)=\left(\frac{1.1019(z-0.5249)(z+0.7494)(z+0.8142)(z-0.1481)}{\left(z-0.3713\right)\left(z+0.6849\right)\left(z+0.7921\right)\left(z+0.07968\right))}\right)$
	$G_{\text{GA}\_2\text{nd}\_6}(z)=\left(\frac{1.1019\left(z-0.01236\right)\left(z-0.3426\right)\left(z-0.5511\right)\left(z+0.7145\right)\left(z+0.7874\right)\left(z+0.8176\right)}{\left(z+0.8069\right)\left(z+0.757\right)\left(z+0.666\right)\left(z-0.4701\right)\left(z-0.1822\right)\left(z+0.1305\right)}\right)$

PSO-GA hybrid optimization	$G_{\text{HYBR}\_2\text{nd}\_2}(z)=\left(\frac{1.1039\left(z-0.5974\right)\left(z+0.7144\right)}{\left(z+0.7261\right)\left(z-0.08744\right)}\right)$
	$G_{\text{HYBR}\_2\text{nd}\_4}(z)=\left(\frac{1.1042\left(z+0.7226\right)\left(z+0.7126\right)\left(z-0.7375\right)\left(z-0.1918\right)}{\left(z-0.5068\right)\left(z+0.7157\right)\left(z+0.7347\right)\left(z+0.08783\right)}\right)$
	$G_{\text{HYBR}\_2\text{nd}\_6}(z)=\left(\frac{1.1042\left(z-0.4648\right)\left(z-0.7778\right)\left(z+0.729\right)\left(z+0.7164\right)\left(z+0.7121\right)\left(z-0.01829\right)}{\left(z-0.6543\right)\left(z-0.238\right)\left(z+0.7136\right)\left(z+0.7213\right)\left(z+0.7384\right)\left(z+0.1412\right)}\right)$

**Table 3:** Mathematical models of half differentiators based on 3rd order optimized operators.

Mathematical models of half differentiators based on 3rd order optimized operators	
Algorithm	Transfer function in pole-zero form
PSO	$G_{\text{PSO}\_\text{3rd}\_3}(z)=\left(\frac{1.0747\left(z-0.7422\right)\left(z+0.3892\right)\left(z+0.2492\right)}{\left(z-0.1853\right)\left(z+0.2354\right)\left(z+0.433\right)}\right)$
	$G_{\text{PSO}\_\text{3rd}\_6}(z)=\left(\frac{1.0748\left(z-0.9033\right)\left(z-0.2948\right)\left(z+0.2512\right)\left(z+0.2397\right)\left(z+0.383\right)\left(z+0.4194\right)}{\left(z-0.6398\right)\left(z+0.4678\right)\left(z+0.3939\right)\left(z+0.2476\right)\left(z+0.223\right)\left(z-0.008066\right)}\right)$
	$G_{\text{PSO}\_\text{3rd}\_9}(z)=\left(\frac{\left(\begin{array}{c} 1.0748\left(z-0.9502\right)\left(z-0.5926\right)\left(z+0.4445\right)\left(z+0.3965\right)\left(z+0.3815\right)\left(z+0.2517\right)\left(z+0.2468\right)\left(z+0.2316\right)\left(z-0.1144\right) \end{array}\right)}{\left(\begin{array}{c} \left(z-0.8073\right)\left(z-0.3441\right)\left(z+0.3865\right)\left(z+0.4144\right)\left(z+0.483\right)\left(z+0.25\right)\left(z+0.2412\right)\left(z+0.2159\right)\left(z+0.04502\right) \end{array}\right)}\right)$

GA	$G_{\text{GA}\_\text{3rd}\_3}(z)=\left(\frac{1.0393\left(z+0.259\right)\left(z-0.2665\right)\left(z-0.4826\right)}{\left(z+0.4564\right)\left(z^{2}-0.2763z+0.2576\right)}\right)$
	$G_{\text{GA}\_\text{3rd}\_6}(z)=\left(\frac{1.0393\left(z-0.824\right)\left(z+0.4138\right)\left(z+0.1912\right)\left(z-0.05418\right)\left(z^{2}-0.3657z+0.244\right))}{\left(z+0.5368\right)\left(z+0.2948\right)\left(z^{2}-0.6608z+0.1659\right)\left(z^{2}-0.1389z+0.27\right))}\right)$
	$G_{\text{GA}\_\text{3rd}\_9}(z)=\left(\frac{\left(\begin{array}{c} 1.0393\left(z-0.8991\right)\left(z+0.4866\right)\left(z+0.3107\right)\left(z+0.163\right)\left(z-0.008699\right)\left(z^{2}-0.6199z+0.1803\right)\left(z^{2}-0.22z+0.2639\right) \end{array}\right)}{\left(\begin{array}{c} \left(z-0.6505\right)\left(z+0.5643\right)\left(z+0.3957\right)\left(z+0.2343\right)\left(z-0.1531\right)\left(z^{2}-0.4068z+0.2363\right)\left(z^{2}-0.1005z+0.2715\right) \end{array}\right)}\right)$

PSO-GA hybrid optimization	$G_{\text{HYBR}\_\text{3rd}\_3}(z)=\left(\frac{1.0761\left(z-0.7193\right)\left(z+0.4597\right)\left(z+0.3395\right)}{\left(z-0.1263\right)\left(z+0.2642\right)\left(z+0.5308\right)}\right)$
	$G_{\text{HYBR}\_\text{3rd}\_6}(z)=\left(\frac{1.0762\left(z-0.8834\right)\left(z-0.2504\right)\left(z+0.2871\right)\left(z+0.3543\right)\left(z+0.4436\right)\left(z+0.5127\right)}{\left(z-0.6142\right)\left(z+0.5698\right)\left(z+0.4702\right)\left(z+0.3298\right)\left(z^{2}+0.2994z+0.02545\right)}\right)$
	$G_{\text{HYBR}\_\text{3rd}\_9}(z)=\left(\frac{\left(\begin{array}{c} 1.0762\left(z-0.9309\right)\left(z-0.5656\right)\left(z+0.5446\right)\left(z+0.4752\right)\left(z+0.4387\right)\left(z+0.3588\right)\left(z+0.3251\right)\left(z+0.2413\right)\left(z-0.0382\right) \end{array}\right)}{\left(\begin{array}{c} \left(z-0.7858\right)\left(z-0.3041\right)\left(z+0.2951\right)\left(z+0.3455\right)\left(z+0.4532\right)\left(z+0.5055\right)\left(z+0.5848\right)\left(z^{2}+0.3459z+0.04169\right) \end{array}\right)}\right)$
